# A Novel Instruction Driven 1-D CNN Processor for ECG Classification

**DOI:** 10.3390/s24134376

**Published:** 2024-07-05

**Authors:** Jiawen Deng, Jie Yang, Xin’an Wang, Xing Zhang

**Affiliations:** 1 The Key Laboratory of Integrated Microsystems, Peking University Shenzhen Graduate School, Shenzhen 518000, China; 2001111225@pku.edu.cn (J.D.); lvdou@stu.pku.edu.cn (J.Y.); 2 School of Integrated Circuits, Peking University, Beijing 100871, China

**Keywords:** R-peak detection, ECG classification, convolutional neural network (CNN), hardware design

## Abstract

Electrocardiography (ECG) has emerged as a ubiquitous diagnostic tool for the identification and characterization of diverse cardiovascular pathologies. Wearable health monitoring devices, equipped with on-device biomedical artificial intelligence (AI) processors, have revolutionized the acquisition, analysis, and interpretation of ECG data. However, these systems necessitate AI processors that exhibit flexible configuration, facilitate portability, and demonstrate optimal performance in terms of power consumption and latency for the realization of various functionalities. To address these challenges, this study proposes an instruction-driven convolutional neural network (CNN) processor. This processor incorporates three key features: (1) An instruction-driven CNN processor to support versatile ECG-based application. (2) A Processing element (PE) array design that simultaneously considers parallelism and data reuse. (3) An activation unit based on the CORDIC algorithm, supporting both Tanh and Sigmoid computations. The design has been implemented using 110 nm CMOS process technology, occupying a die area of 1.35 mm^2^ with 12.94 µW power consumption. It has been demonstrated with two typical ECG AI applications, including two-class (i.e., normal/abnormal) classification and five-class classification. The proposed 1-D CNN algorithm performs with a 97.95% accuracy for the two-class classification and 97.9% for the five-class classification, respectively.

## 1. Introduction

Cardiovascular diseases (CVDs), including myocardial infarction, cardiac arrhythmia, cardiomyopathy, and myocarditis, account for 31% of global mortality, as reported by the World Health Organization (WHO) [1,2]. Among the various chronic CVDs, cardiac arrhythmia stands out as the most prevalent, resulting in the highest number of fatalities attributed to cardiac arrests. One of the most effective tools for diagnosing heart diseases is the electrocardiogram (ECG), a noninvasive technique that records the fluctuation of the heart’s bio-electric activities.

However, existing wearable ECG devices currently rely heavily on cloud platforms for disease diagnosis, necessitating the transmission of raw ECG data from the device to a remote setup via mobile phones or the Internet [3]. This causes high power consumption and delayed diagnosis, hindering prompt treatment. Consequently, there is an urgent need to develop wearable healthcare devices capable of providing high-precision medical diagnoses to address this issue.

The classification of ECG signals typically involves a combination of pre-processing, feature extraction, and classification algorithms [4]. The raw ECG signal may be contaminated by various sources of noise. Therefore, pre-processing techniques such as digital filter hardware design techniques [5,6,7] help to denoise the ECG. In terms of classification, conventional machine learning methods such as Support Vector Machine (SVM) [8,9,10], and K-Nearest Neighbors (KNN) [11,12] operate by classifying the time/frequency-domain feature extracted by the feature extraction step from the input signals. However, the classification accuracy largely relies on the quality of the manually selected features, which is limited by the experience of the designer. Furthermore, feature engineering requires significant design efforts and poses challenges for hardware implementation in terms of resource consumption reduction. 

Unlike conventional machine learning methods, neural networks, specifically convolutional neural networks (CNNs) [13,14,15,16], do not require the design of complex feature extraction algorithms as they automatically extract high-level features from the input and perform classification simultaneously. Therefore, CNN-based methods demonstrate exceptional classification and prediction capabilities across a vast spectrum of diverse databases.

Kiranyaz Serkan et al. [17] proposed an adaptive implementation of 1-D CNNs for real-time classification of patient-specific ECG signals. By utilizing 1-D CNNs, the two main components of feature extraction and classification in traditional ECG classification are fused into a single step, negating the need to extract manual crafted features. Tesfai Huruy et al. [18] presented a lightweight 1-D CNNs considering channel shuffle over the group and depth-wise convolutions, which takes 2-s ECG signal segments as input. The proposed model outperformed traditional CNNs with 9× fewer trainable parameters and improved the F1-score by 2%.

For ECG classification using 2-D CNNs, a 2-D transformation must be applied to make the time series suitable for the image-like 2-D input. Amin Ullah et al. [19] transformed the 1D ECG recordings to 2D gray-scale images with a size of 512 × 512, which were then fed as input for the 2D CNN for the detection of five types of arrhythmias. GYU-HO CHOI et al. [20] proposed a user recognition system that conducts 2D feature extraction by converting the P waves, QRS complexes, and T waves of a single period of a 1D ECG signal into a spectrogram, resizes the spectrogram into 2D using a bi-cubic interpolation method, and then applies classification for user recognition. Wenhan Liu et al. [21] proposed a multilead-CNN model including sub 2D convolutional layers and lead asymmetric pooling layers, taking multilead ECG as input to detect myocardial infarction.

In general, 1-D CNNs are often preferred over 2-D CNNs for real-time ECG classification due to their simpler operation, higher computation speed, and fewer learnable parameters. This makes 1-D CNNs more suitable for real-time ECG classification and easier to implement in hardware.

However, the end-to-end neural network incurs high computational complexity due to its multi-layered architecture that carries huge numbers of neurons (computing elements), resulting in significant latency and energy consumption. Hence, application-specific dedicated hardware optimization techniques are required for ECG classification. Additionally, in contrast to 2-D CNNs, the convolutional kernels in 1-D CNNs only need to slide in one direction along the input features. Consequently, existing 2-D CNN accelerators are not as effective as 1-D CNN. 

Furthermore, the reduced control complexity presents an opportunity to explore more efficient data reuse and computational parallelism in 1-D CNN accelerator hardware design. Currently, there are ECG classification processors based on 1-D CNNs. However, these architectures are specifically tailored to a particular 1-D CNN structure, limiting their flexibility and hindering subsequent algorithm upgrades.

Hence, this work focuses on building a 1-D CNNs accelerator hardware architecture for arrhythmia detection and quick diagnosis while maintaining acceptable flexibility for more potential application. This work aims to optimize the hardware in three key aspects: reducing computation time, memory access time, and memory footprint to achieve a better trade-off between area, accuracy, energy, latency, and computational complexity.

The contributions of this work are as follows:

1. An instruction driven 1-D CNN accelerator that allows for flexible configuration of CNNs architecture and eliminates the intermediate data transfer by memory access between the convolution layer and the activation layer, as well as the activation layer and the pooling layer.
2. A process element (PE) array with a 1D arrangement that exploits the parallelism in inter-kernel and intra-kernel patterns and data reuse in row-stationary (RS) dataflow.
3. A CORDIC-based module, utilizing the FP16 data format, is employed for the computation of the Tanh and Sigmoid activation functions.

The rest of this paper is organized as follows. Section 2 presents an overview of the characteristics of ECG signals, provides essential information about the dataset used, and introduces the background knowledge on CNNs. Section 3 presents the overall architecture of the processor, including the detailed architecture and design considerations of each module. Section 4 shows the results of algorithm testing and chip measurements, along with a comparison to previous works. Finally, Section 5 concludes the study.

## 2. Background

### 2.1. ECG Signals and Database

**Figure 1:**
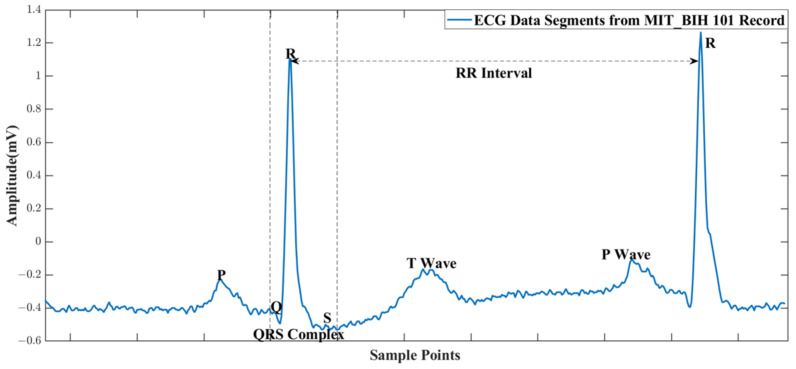
ECG waveform from MIT_BIH database.

ECG is a widely used diagnostic tool in the field of cardiology, allowing healthcare professionals to assess the electrical activity of the heart. The ECG waveform consists of recognizable components, as shown in Figure 1, including the P wave, QRS complex, and T wave, each representing a specific phase of the cardiac cycle. Changes in the amplitude, duration, or morphology of these components can indicate underlying cardiac abnormalities.

The original ECG signals used in this paper are provided by the MIT-BIH database [22]. This database contains 48 records of heartbeats with a sampling rate of 360 Hz with an 11 bit resolution for approximately 30 min of data from 47 different patients. Each record comprises two ECG leads, with the primary lead being a modification of lead II, which serves as experiment data in this paper. The secondary lead is typically a modified version of lead V1, but in some cases, it may be V2, V5, or V4.

According to the standard developed by the Association for the Advancement of Medical Instrumentation (AAMI) [23], the heartbeat types that exist in the MIT-BIH database are grouped into five different classes: Normal (N), Supraventricular ectopic beat (SVEB), Ventricular ectopic beat (VEB), Fusion beat (F) and Unknown beat (Q), which consists of 17 subcategories, as shown in Table 1.

In this paper, we developed two 1D-CNN models and corresponding instructions for two-class (i.e., normal/abnormal) classification and five-class classification following the AAMI standard to demonstrate the flexibility of the processor.

### 2.2. Convolutional Neural Networks

An artificial neural network is a computational model inspired by the structure and functioning of the human brain. It comprises interconnected artificial neurons, analogous to the interconnected nerve cells in the human nervous system that communicate through synapses. Within the realm of artificial neural networks, CNNs are a specific type that has demonstrated remarkable effectiveness in various tasks related to image and signal processing. 

CNNs generally consists of four types of layers: convolution layer, pooling layer, activation layer, and fully connected layer. Among them, the convolutional layer and the fully connected (FC) layer stand out as having the highest computational complexity. Qiu et al. [24] conducted an analysis of the computational and memory requirements associated with convolution and FC layers. Their findings revealed that convolution layers exhibit a high degree of computational intensity, while FC layers demonstrate a significant demand for memory resources.

### 2.3. Running Example: 1-D Convolution Layer Optimization Overview 

**Figure 2:**
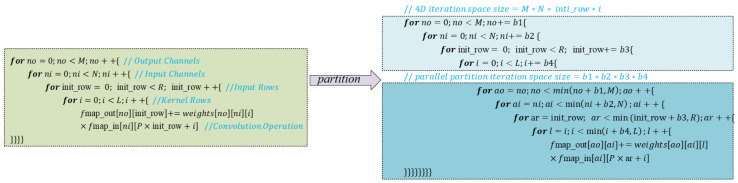
Algorithm for 1-D convolution layer and partition.

Figure 2 presents the code implementation of a 1-D convolution layer, which employs four nested loops. In 1-D convolution, the filter moves unidirectionally, and both the input feature map and the convolution operation are one-dimensional. The outermost loop iterates through all the output feature maps (fmap_out[]). Each specific output feature map is computed as the summation of convolutions performed between each input feature map and its corresponding convolution kernel. It is important to note that the computational complexity of evaluating the deeply nested four loops can be significantly high, depending on the size of each layer. Consequently, parallelizing the computation of all the output feature maps becomes crucial to mitigate the computational burden.

The parallelization can be accomplished through two distinct approaches: either mapping the partitions generated by the outermost four loops to different processing elements (PEs) or assigning the convolution of each partition (innermost four loops) to different PEs. An essential consideration in this domain is determining the specific values of *b*1 … *b*4. This decision carries implications regarding data dependencies among the PEs, data communication requirements, and the achievable level of parallelism, which leads to different parallelization strategies, considering storage limitations and data communication. Currently, there are three kinds of parallelism: inter-output, inter-kernel, and intra-kernel. 

In this paper, we employ a MAC group design, where each MAC group consists of multiple MAC units, which enables simultaneous computation of multiply-accumulate operations, facilitating intra-kernel parallelism. Additionally, four MAC groups are employed to simultaneously compute different elements within the same output feature map, further contributing to inter-kernel parallelism.

Furthermore, the parallelism approach determines the data access pattern, and thus different parallelization approaches result in different data flows. To minimize memory access time, we explore the row-stationary (RS) dataflow which reuses all the types of data: weights, activations and partial sums by leveraging on-chip data and weight buffers, along with direct data transfer between convolutional and activation layers, as well as between activation and pooling layers.

## 3. Hardware Design

### 3.1. Overall Architecture

**Figure 3:**
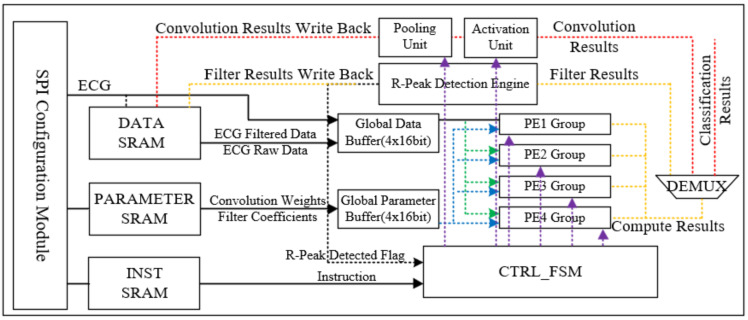
Block diagram of the proposed system.

Figure 3 shows the overall architecture of the proposed 1-D CNN processor. The processor primarily consists of three components, each responsible for data storage, R-peak detection, and CNN inference, respectively. First, the PE array, instruction control FSM, activation unit, and pooling unit are used to perform CNN inference. Second, the R-peak detection engine is responsible for R-peak detection and ECG signal segmentation. Third, there is a SRAM array, which includes instruction storage, parameter storage, data storage, and the corresponding SPI configuration module.

The classification model training is performed offline. A customized compiler is designed to convert the PyTorch 1-D CNN model to the instructions automatically. Then the SPI configuration module loads the parameters of 1-D CNN, the filter coefficients and the corresponding 1-D CNN instructions. 

**Figure 4:**
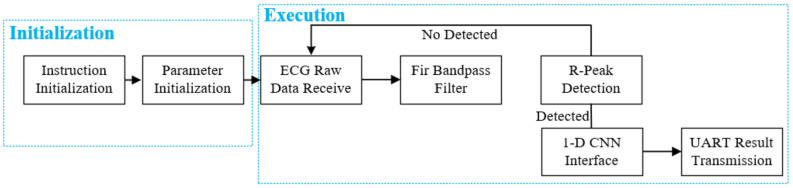
Work flow of the proposed system.

The analog front-end for ECG acquisition and digitization is implemented using TI’s ADS1232 and is connected to the processor via the SPI interface. For every ECG data received by the SPI interface is processed in the processor as illustrated in Figure 4, starting with the FIR bandpass filter reusing the PE array, then the PAT algorithm-based R-peak detection engine. When an R-peak is detected, the 1-D CNN interface will be executed with a 300-sample ECG segment as the input feature, 100 samples before the R peak and 199 samples after the R peak. Upon completion of the computation, the results are transmitted via the UART interface.

### 3.2. ECG Pre-Processing Engine

#### 3.2.1. Denoise

ECG recordings are typically contaminated by various types of noise and artifacts. During the denoising process, the objectives are to reduce such noise and artifacts, and prevent amplitude and offset effects when comparing signals from different patients. Bandpass filtering is widely used to eliminate muscle noise, baseline wander, power line interference, and low- and high-frequency noise components, as well as to limit ADC saturation and address antialiasing.

In this paper, we employed a bandpass filter based on Finite Impulse Response (FIR) with a frequency range of 0.5–40 Hz, chosen based on the majority’s preference [25]. We observed that the calculation method of FIR and one-dimensional convolution with a stride of 1 is highly similar. Therefore, we did not use a specialized FIR filter calculation module, but instead reused the instruction-based 1-D CNN calculation engine introduced in the next chapter to reduce hardware overhead.

#### 3.2.2. R-Peak Detection Engine

The detection engine in this study implements the widely used Pan–Tompkins algorithm. First, the QRS waves centered at 10 Hz are retrieved, and the P and T waves are attenuated through cascaded low-pass and high-pass filters. It is crucial to retain the information carried by the ECG signal after filtering. The difference equations of the cascaded low-pass filter (LPF) and high-pass filter (HPF) filters are shown in Equations (1) and (2), respectively.

(1) $$y\left(nT\right)=2y\left(nT-T\right)-y\left(nT-2T\right)+x\left(nT\right)-2x\left(nT-6T\right)+x(nT-12T),$$

(2) $$y\left(nT\right)=x\left(nT-16T\right)-x\left(nT-17T\right)+y(nT-T)-\frac{1}{32}[x\left(nT\right)-x\left(nT-32T\right)]$$

**Figure 5:**
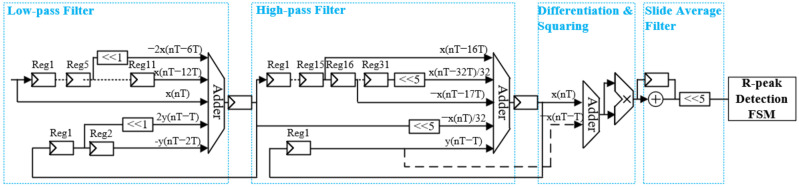
Hardware architecture of the R-peak detection engine.

The LPF filter has a cutoff frequency of 11 Hz and introduces a delay of 12 samples, while the HPF filter has a cutoff frequency of 5 Hz and a delay of 32 samples. This paper implements the following formula using only shift registers, shifters, and adders to reduce hardware costs as shown in Figure 5.

After the filtering process, the next step involves differentiating the filtered signal to distinguish the QRS complex from other ECG waves by finding high slopes. Subsequently, a non-linear transformation is applied by squaring the differentiated ECG signal point-by-point. This step is crucial for highlighting the higher frequencies in the signal that are typically associated with the QRS complex. After that, the QRS waves are smoothed with the 32-order sliding average filter. Finally, adaptive amplitude thresholds are applied to the averaged signal to detect R peaks through R-peak detection FSM.

After the R-peak is located, a refractory period of 200 ms is established to prevent false detection caused by the subsequent T-wave. Furthermore, to further enhance the accuracy of R-peak detection, the FSM searches for the maximum value within a window of ±25 samples around the potential R-peak from the data buffer.

After the detection of the R-peak, the FSM calculates the starting address of the 100 samples preceding the R-peak and transfers it to the INST_CTRL_FSM module. Subsequently, upon receiving the 199 samples following the R peak, a signal is assigned to control the start of 1-D CNN inference.

### 3.3. 1-D CNN Engine

#### 3.3.1. Overall Architecture

**Figure 6:**
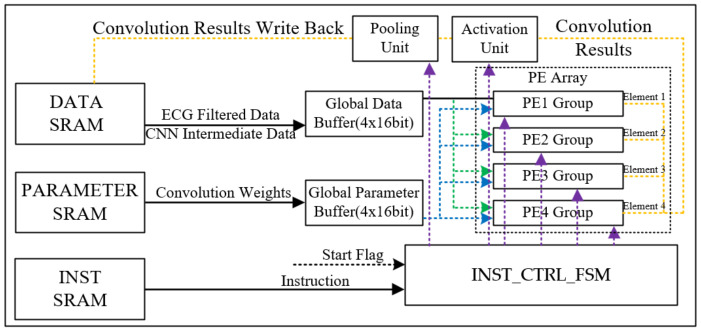
The overall architecture of the 1-D CNN engine.

The 1-D CNN engine primarily consists of data and parameter buffers, a PE array, as well as activation and pooling units as shown in Figure 6. The utilization of on-chip buffers aims to enhance data utilization, thus reducing the number of memory accesses through the temporal reuse of weight and data.

To reduce computational time and facilitate parallel processing, this research introduces a distinctive PE array. The PE array comprises four MAC groups, and each group consists of four multiply-accumulate (MAC) units to enable the concurrent parallelization of inter-kernel and intra-kernel operations.

The activation units utilized in this study support the rectified linear unit (ReLU), hyperbolic tangent (Tanh), and Sigmoid activation functions. Additionally, in order to ensure compatibility with the previously mentioned FIR filter, a bypass mode is supported. As for the pooling units, both max pooling and average pooling are supported, also with a bypass mode. All of these configurations are controlled and implemented through instruction decoded by the INST_CTRL_FSM module.

#### 3.3.2. 1-D CNN Instruction

The instruction format utilized by the processor is depicted in Table 2, which describes the structure of convolutional layers and fully connected layers (e.g., the type of layers, size of input/output feature map, size of kernel, stride size, activation function). The data width of instruction SRAM is 16 bit, so the INST_CTRL_FSM needs six clock cycles to fetch and decode the instructions of each 1-D CNN layer, and the subsequent instruction is fetched only after the completion of the current instruction.

#### 3.3.3. PE Array and MAC Unit

**Figure 7:**
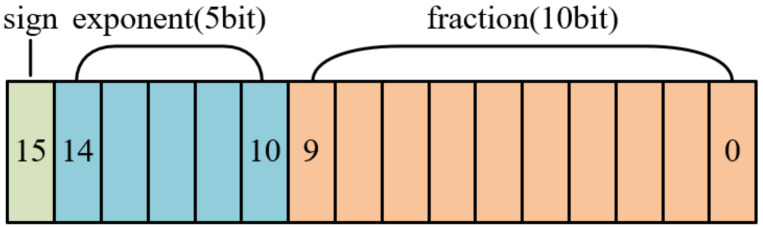
The IEEE 754 standard half-precision floating-point data format.

In pursuit of reduced memory footprint, enhanced energy efficiency, and facilitated post-training model quantization, this processor adopts an FP16 data format for calculation. The FP16, as shown in Figure 7, also known as half-precision floating-point, is a data type used to represent floating-point numbers using 16 bits (2 bytes). It offers a compromise between the precision of FP32 (single-precision) and the memory efficiency of FP8 (half-precision).

To perform computations on FP16 data, this paper presents a MAC unit which uses a Vedic multiplier and a carry-lookahead adder for efficient multiplication and addition operations. Additionally, the MAC unit incorporates an enable signal that allows for idling when not required, thereby reducing power consumption. Furthermore, the MAC unit generates an error signal to indicate overflow and underflow conditions.

**Figure 8:**
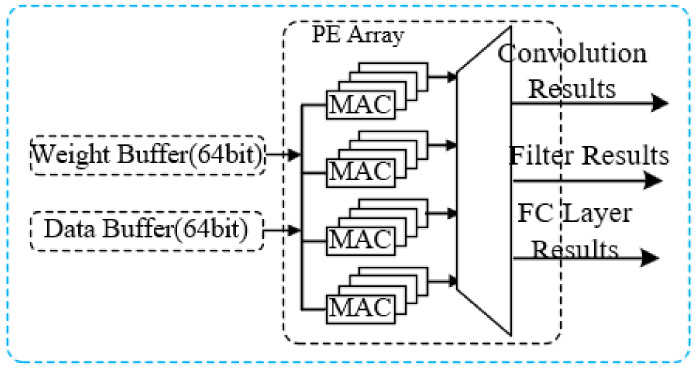
The demonstration of the PE array and data buffer.

Four MAC units are grouped together to form a MAC group, and four MAC groups are arranged to form a processing element (PE) array as shown in Figure 8. The PE array serves as the computational engine for both convolutional and fully connected layers. During convolutional computations, each MAC group is assigned the task of generating a single output element. Within each MAC group, all MAC units concurrently compute the partial sums required for the corresponding output element.

To optimize data utilization, input feature maps and weight parameters are broadcasted via on-chip buffers. Following the computation phase, the resultant outputs from each MAC group are channeled through a multiplexer module. This module, guided by instructions, distributes the outputs into distinct data streams corresponding to the results of the convolutional layer, the FIR filter, and the fully connected layer.

#### 3.3.4. Activation Unit

Activation functions such as the Rectified Linear Unit (ReLU), Sigmoid, and the Hyperbolic Tangent (Tanh) constitute an integral component of neural network architectures, playing a pivotal role in determining the network’s capacity to model complex relationships within data. The selection of activation function can significantly impact the performance and efficiency of a neural network. Different network architectures often necessitate the use of distinct activation functions to achieve optimal results. Consequently, the development of reconfigurable hardware capable of supporting a diverse range of activation functions has become a critical requirement in the field of neural network implementation.

The hardware implementation of the ReLU activation function is relatively simple, while it is more challenging for the other two. In this paper, instead of using the area-consuming look-up table (LUT)-based approach, we adopted the CORDIC algorithm to compute the Sigmoid and Tanh functions.

Mathematically, the definition formulas for Tanh and Sigmoid, as well as their relationship with the function, are shown in Equations (3)–(5).

(3) $$Sigmoid\left(x\right)=\frac{1}{1+e^{-x}\;},$$

(4) $$Tanh\left(x\right)=\frac{e^{x}{-e}^{-x}}{e^{x}+e^{-x}}\;,$$

(5) $$2Sigmoid\left(2x\right)-1=\frac{2}{1+e^{-2x}}-1=\frac{1-e^{-2x}}{1+e^{-2x}}=Tanh(x)$$

**Figure 9:**
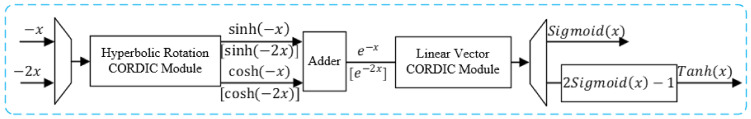
The architecture of computing Sigmoid(x) and Tanh(x) and T(x) based on CORDIC.

Drawing upon the mathematical foundations established by the above equations, we design the architecture depicted in Figure 9 to calculate Sigmoid(x) or Tanh(x).

This architecture comprises two principal components: a CORDIC iteration module operating in the rotation mode of the hyperbolic coordinate system, and a CORDIC iteration module operating in the vector mode of the linear coordinate system. The first CORDIC module computes the hyperbolic sine (sinh) and hyperbolic cosine (cosh) functions, while the second CORDIC module performs division operations.

The traditional input range for the first module is limited to |x| ≤ 1.1182. The second module, however, has a constraint of $|\frac{y}{x}|\;\leq \;1$. Since the ranges of both Sigmoid(x) and Tanh(x) functions satisfy this requirement, we do not need to be concerned about the input limitations of the second module. To accommodate different input ranges for the first module, we modified the iteration index set from k = 0, 1, …, n to k = −m, −m + 1, …, 0, 1, …, n, based on the approach described in [26] to expand the range of input variables *x* of the first module.

#### 3.3.5. Pooling Unit

CNNs employ a pooling layer to downsample the feature maps generated by the convolution layers. This process not only adds non-linearity but also decreases the dimensionality of the input data, thereby minimizing the computational burden of subsequent network layers and expediting the overall inference process. Two commonly used pooling methods are average pooling and max pooling.

**Figure 10:**
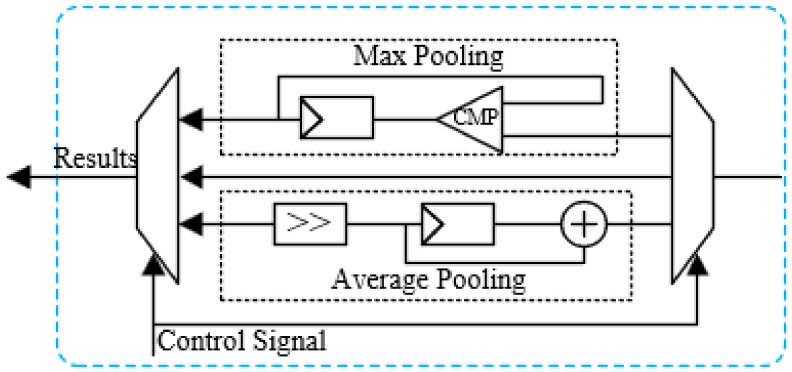
The architecture of the pooling unit.

To accommodate both average and max pooling operations, this paper proposes a pooling unit, depicted in Figure 10, primarily composed of comparator, shifter, adder and registers. In pursuit of reduced power consumption and a minimized area, the proposed design leverages shift operations to implement division in average pooling. Control signals are generated by the INST_CTRL_FSM module to control the pooling type and the size of the pooling filter.

## 4. Results and Discussion

### 4.1. Software Implementation Results

To demonstrate the configurability of the proposed architecture and evaluate its performance and power consumption, we designed two 1D-CNN models for two-class (i.e., normal/abnormal) classification and five-class classification following the aforementioned AAMI standard, respectively. The FC layer is responsible for performing classification; therefore, the neurons number of the output should correspond to the number of result categories. Model development and training was carried out using the PyTorch framework. After training was completed, the model parameters were converted to FP16 by quantization. Thanks to the broader data representation range of FP16, the quantized model experiences only a slight loss in performance. The detailed structures of the two 1D-CNN models are shown in Table 3 and Table 4.

Utilizing the MIT-BIH database, we obtained their performance metrics, including accuracy, sensitivity, and specificity, which are tabulated in Table 5 and Table 6. Two types of accuracy are reported: (1) individual class accuracy for a specific class, and (2) the overall accuracy, measured as the ratio of the sum of diagonal elements to the sum of all elements in the confusion matrix.

### 4.2. ASIC Implementation Results

The 1-D CNN-based processor is implemented in the HHGrace 110 nm 1P6M CMOS process. The design was synthesized by using the Synopsys Design Compiler, and then floor planned, placed, and routed by using Cadence Innovus. The provided results are based on a post-layout power and area analysis.

**Figure 11:**
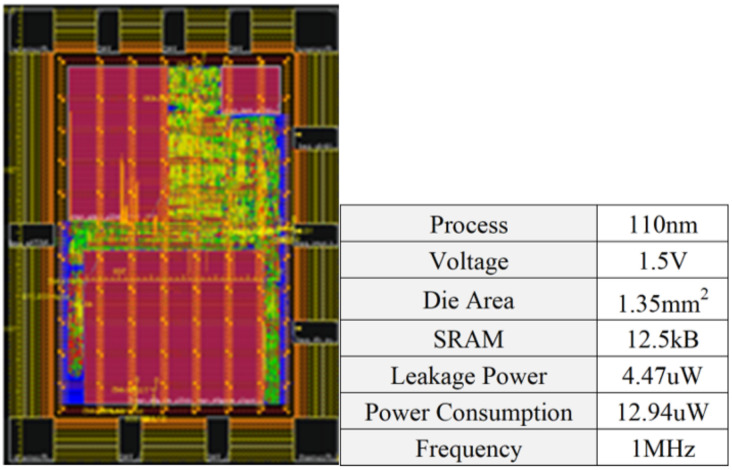
The processor layout and specifications.

Figure 11 shows the floorplan of the design. The placed and routed processor footprint dimensions were 1380 µm by 980 µm with a total utilization of 81.2%. The design occupied a total core area of 1.352 mm^2^. It integrates 12.5 KB SRAM for data and instruction storage. The leakage power is 4.47 μW at 1.5 V power supply voltage. The total power for the ECG classification is 12.94 μW at 1 MHz.

### 4.3. Comparison

The comparison of the proposed processor with the state-of-the-art biomedical processor for ECG classification is shown in Table 7 validated with the same MIT-BIH database.

As reported in [27], the SVM algorithm employed for classification requires the storage of a large number of support vectors, incurring significant memory overhead. The authors of [28,29,30] leverage specialized forms of neural networks. Specifically, [30] employs an Extreme Learning Machine (ELM), which is a type of single-hidden-layer feed-forward neural network; this simplistic network architecture results in suboptimal classification performance. Amongst the referenced works [28,29], the processor presented in [29] exhibits superior energy efficiency characteristics. This can be attributed to the use of lower operating frequency and supply voltage, coupled with the adoption of a Ternary Neural Network (TNN) architecture for classification. However, the adoption of the TNN architecture introduces challenges in terms of model development and quantization. Additionally, both approaches leverage fixed network structures, which constrains their applicability and versatility across diverse use cases. The processor proposed in this work demonstrates comparable accuracy and energy efficiency performance relative to the state-of-the-art prior implementations. Notably, its inherent configurability enables adaptation to diverse application, unlike other designs that are restricted to a single task.

## 5. Conclusions

This work focuses on designing a reconfigurable AI processor for ECG-based applications, aiming to achieve low power consumption and high accuracy. The proposed processor leverages diverse architectural and circuit techniques to optimize the trade-off between area, accuracy, energy, latency, and computational complexity. These techniques include an instruction-driven AI processor to support versatile 1D CNN processing, a PE array design that simultaneously considers parallelism and data reuse to maximize computational throughput and reduce memory access time, an activation unit based on the CORDIC algorithm, supporting both Tanh and Sigmoid computations with low hardware complexity.

The processor is implemented using 110 nm CMOS process technology with FP16 data format for easy quantization. Its performance is demonstrated on two typical ECG classification applications, achieving 97.8% and 93.5% accuracy for two-class and five-class classification, respectively. The design is validated on the MIT-BIH database, but further research is needed to address real-world variations in patient data. Future work will focus on developing an adaptive learning engine to improve classification accuracy and personalize the system for individual users.

Despite the limitations of the current implementation, the underlying conceptual framework of the proposed AI processor remains highly valuable. Its inherent flexibility allows for adjustments in the instruction to accommodate the execution of new CNN algorithms tailored to specific applications and datasets. This adaptability, coupled with the efficient instruction-driven architecture, renders the proposed AI processor a practical and effective solution for real-time ECG classification on wearable devices, paving the way for further advancements in AI-powered healthcare technologies.

## Figures and Tables

**Table 1:** The correspondence between the AAMI heartbeat classes and the heartbeat types in the MIT-BIH database.

AAMI Beat Class	Symbol	MIT-BIH Normal/Arrhythmia Types	Symbol
Non-ectopic beats	N	Normal Beats	N
		Left bundle branch block	L
		Right bundle branch block	R
		Atrial escape beats	e
		Nodal (junctional) escape beats	j
Supraventricular ectopic beats	SVEB	Atrial premature beat	A
		Aberrated atrial premature beat	a
		Nodal (junctional) premature beat	J
		Supraventricular premature beat	S
Ventricular ectopic beat	VEB	Premature ventricular contraction	V
		Ventricular escape beat	E
Fusion beat	F	Fusion of ventricular and normal beat	F
Unknown beat	Q	Paced beat	P/
		Fusion of paced and normal beat	f
		Unclassifiable beat	U

**Table 2:** The instruction format for the proposed processor.

Instruction Field	Width	Represent
Layer Type	1 bit	0: Conv Layer; 1: FC Layer
Input Row	10 bit	Number of input feature’s row
Input Channel	8 bit	Number of input feature’s channel
Input Data SRAM ADDR	12 bit	Address of data stored in Data SRAM
Kernel Row	5 bit	Number of kernel’s row
Output Channel	8 bit	Number of output feature’s channel
Kernel Weight SRAM ADDR	13 bit	Address of weight stored in Weight SRAM
Conv Stride	2 bit	Convolution Operation Stride
Activation Function	2 bit	00: Bypass 01: Relu 10: Sigmoid 11: Tanh
Pool Type	1 bit	00: Maxpooling 01: Avgpooling 10: Bypass
Pool Size	3 bit	Size of pooling operation
Output Data SRAM ADDR	12 bit	Address of output data stored in data SRAM

**Table 3:** The 1-D CNN model for ECG two-class classification.

Layer	Parameter
Conv1 + Tanh1 + Maxpooling1	Kernel_size = 1 × 16 × 12 Pooling_Size = 4
Conv2 + Tanh2 + Maxpooling2	Kernel_size = 16 × 32 × 12 Pooling_Size = 4
FC	480-5

**Table 4:** The 1-D CNN model for ECG five-class classification.

Layer	Parameter
Conv1 + ReLu1 + Maxpooling1	Kernel_size = 1 × 16 × 12 Pooling_Size = 4
Conv2 + ReLu2 + Maxpooling2	Kernel_size = 16 × 32 × 8 Pooling_Size = 2
Conv3 + ReLu3 + Maxpooling3	Kernel_size = 32 × 32 × 4 Pooling_Size = 2
FC	448-5

**Table 5:** Performance parameters of the proposed 1-D CNN model over the entire database for two-class classification.

	Normal	Abnormal
Acc (%)	97.95%	97.95%
Sen (%)	98.76%	95.99%
Spe (%)	95.99%	98.76%
+P (%)	97.89%	97.00%
Overall Acc (%)	97.95%	

**Table 6:** Performance parameters of the proposed 1-D CNN model over the entire database for five-class classification.

	N	SVEB	VEB	F	Q
Acc (%)	98.10%	98.93%	99.12%	99.69%	99.90%
Sen (%)	99.44%	61.72%	93.84%	71.40%	98.97%
Spe (%)	90.15%	99.82%	99.56%	99.93%	99.94%
+P (%)	98.36%	89.17%	94.63%	90.51%	98.72%
Overall Acc (%)	97.90%				

**Table 7:** Comparison with the state-of-the-art ECG processor.

	This Work	[27]	[28]	[29]	[30]
**Technology (nm)**	110	28	180	65	40
**Supply Voltage (V)**	1.5	0.4/0.5/0.8	1.8 V	0.9	0.9
**Classifier**	CNN	SVM	ANN	TNN	AE_ELM
**ECG Classification Task**	2/5 classes	3 classes	5 classes	13 classes	2 classes
**SRAM Size (kB)**	12.5	96	64	1	NA
**Area (mm^2^)**	1.35	0.024	0.92	1.938	0.2123
**Accuracy (%)**	97.95 (2-classes) 97.90 (5-classes)	91.4	98	99.3	92
**Power Consumption (uW)**	12.94	13.1	13.34	1.68	19,164
**Energy Efficiency (uJ/class)**	3.34/5.72	2.1	3.21	0.072	477.3

## Data Availability

Data are contained within the article.
